# Effect of enhanced homestead food production on anaemia among Cambodian women and children: A cluster randomized controlled trial

**DOI:** 10.1111/mcn.12757

**Published:** 2019-05-31

**Authors:** Kristina D. Michaux, Kroeun Hou, Crystal D. Karakochuk, Kyly C. Whitfield, Sokhoing Ly, Vashti Verbowski, Ame Stormer, Keith Porter, Kathy H. Li, Lisa A. Houghton, Larry D. Lynd, Aminuzzaman Talukder, Judy McLean, Timothy J. Green

**Affiliations:** ^1^ Food, Nutrition and Health University of British Columbia Vancouver British Columbia Canada; ^2^ Helen Keller International New York NY USA; ^3^ Department of Applied Human Nutrition Mount Saint Vincent University Halifax Nova Scotia Canada; ^4^ Faculty of Pharmaceutical Sciences University of British Columbia Vancouver British Columbia Canada; ^5^ Department of Human Nutrition University of Otago Dunedin New Zealand; ^6^ Centre for Health Evaluation and Outcome Sciences Providence Health Research Institute Vancouver British Columbia Canada; ^7^ Department of Pediatrics and Reproductive Health University of Adelaide Adelaide South Australia Australia; ^8^ Healthy Mothers, Babies, Children Research Theme South Australia Health and Medical Research Institute, Women's and Children's Hospital Adelaide South Australia Australia

**Keywords:** anaemia, Cambodia, enhanced homestead food production, fishponds, nutrition sensitive, women of childbearing age

## Abstract

There is inconsistent evidence on the efficacy of agriculture programmes at improving women and children's anaemia and nutritional status. The primary aim of this study was to evaluate the impact of a nutrition‐sensitive enhanced homestead food production (EHFP) programme on anaemia in women (18–45 years) and children (6–59 months) in rural Cambodia. Secondary outcomes were women's micronutrient status and women and children's anthropometry. In this cluster‐randomized controlled trial, 900 households from 90 villages (clusters) were randomized to either (a) home gardens and behaviour change communication (BCC) on nutrition, hygiene, women's empowerment, and marketing (EHFP); (b) home gardens plus fishponds and BCC (EHFP + F); or (c) control (no intervention). Haemoglobin concentration and anthropometry were measured in women and children at baseline and at 22 months. Venous blood samples were collected in a subset of women *(n =* 450) at baseline and at 22 months. Generalized linear mixed effect models with repeated measures were used to evaluate the difference across groups and the change from baseline to end of study. Ninety clusters, 552 women, and 754 children completed the trial. Compared with control, we found a statistically significant impact on anaemia prevalence in children (−14.0 percentage points; *P* = 0.02) and retinol binding protein concentrations in women (difference in difference: 0.34; *P* = 0.02) randomized to EHFP and EHFP + F groups, respectively. No other statistically significant effects on anaemia, nutritional biomarker concentrations, or anthropometry were observed. Future research is needed to examine longer term impacts of EHFP on anthropometry in women and children and into the nutritional causes of anaemia among children in Cambodia.

**Key messages**

- This research shows a significant change from baseline to 22 months in anaemia rates among children and RBP concentration in women randomized to the EHFP and EHFP + F groups, respectively.
- Prevalence of micronutrient deficiencies associated with anaemia, particularly iron and vitamin A, are low in women living in Prey Veng, Cambodia, thus highlighting the importance of ensuring a clear understanding of the underlying nutritional deficiencies in a population prior to commencing an intervention.
- The full impact of nutrition‐sensitive agriculture programmes on the nutritional status, particularly anthropometry and micronutrient status, of women and children should be explored for much longer duration; studies ought to include women prior to pregnancy and through the first 1,000 days, at minimum.

## 1 INTRODUCTION

Undernutrition remains a major problem in Cambodia, especially among poor rural women subsistence farmers and their young children. In the most recent Cambodia Demographic and Health Survey (DHS) (2014), over 30% of young children were stunted, and 24% and 14% of children and women of reproductive age (WRA) were underweight, respectively (National Institute of Statistics Ministry of Planning, 2015). Likewise, anaemia has continued to be an intractable disease, with similar rates observed in young children (56%) and women (45%; National Institute of Statistics Ministry of Planning, 2015). There is high food insecurity and low dietary diversity with polished rice providing up to 65% of total energy (Maltsoglou, Dawe, & Tasciotti, 2010). To address these issues, Helen Keller International (HKI) has implemented a programme of enhanced homestead food production (EHFP) in Cambodia, and elsewhere. HKI's EHFP programme focuses on increasing and diversifying plant‐based agriculture production (homestead gardens) and animal production, such as chickens. The programme provides initial farming inputs coupled with technical assistance, nutrition and hygiene behaviour change communication (BCC), and education and training on marketing and gender equity (Olney, Talukder, Iannotti, Ruel, & Quinn, 2009). For the current study, because fish plays an important role in the traditional Cambodian diet, the inclusion of small homestead fishponds that allows small and large fish (polyculture), a high‐quality source of protein and micronutrients, to be produced for household consumption or sale, was considered be an optimal addition to plant‐based EHFP, instead of the traditional programme of small‐scale poultry production.

The programme impact pathways for EHFP have been well described (Haselow, Stormer, & Pries, 2016; Osei et al., 2017); briefly, they postulate that by adopting EHFP activities, agricultural production will increase, including nutrient‐dense animal foods, thereby diversifying the diet, increasing energy, protein and micronutrient intake, and ultimately improving nutritional status (Haselow et al., 2016; Masset, Haddad, Cornelius, & Isaza‐Castro, 2012). Additionally, agricultural production can be sold for income that can then be used to purchase high‐quality, nutritious foods, or health/educational products. Another pathway is through intensive BCC on essential nutrition and hygiene practices and gender equity. By providing all family members with training on women's empowerment and much‐needed nutrition information to caregivers (including mothers, fathers, and grandparents) and linking this knowledge to the new foods being produced by households, the consumption of nutrient‐dense foods will increase, and nutrition and feeding practices will improve for both women and children (Haselow et al., 2016).

Although EHFP has been used in many settings, there is still inconsistent evidence of its efficacy in improving nutritional outcomes (Girard, Self, McAuliffe, & Olude, 2012; Masset et al., 2012; Ruel, & Alderman, 2013). In 2012, two systematic reviews were conducted to determine whether agricultural interventions improve nutritional status of women and children, and both concluded that the question could not be answered with any level of confidence and called for more rigorous studies (Girard et al., 2012; Masset et al., 2012). Since then, there have been two cluster‐randomized control trials examining the impact of HKI's EHFP programme on anaemia and anthropometry; the first one was carried out in Burkina Faso, and the other, more recent, was conducted in Nepal (Olney et al., 2016; Olney, Pedehombga, Ruel, & Dillon, 2015; Osei et al., 2017). In the former, 55 villages were randomized to a control group (*n* = 25) or one of two EHFP groups (*n* = 15 each), which differed only by who delivered the BCC messages (Olney et al., 2015). Women's dietary diversity scores improved (0.3 ± 0.2; *P* = 0.08) and the prevalence of underweight women decreased over 22 months (−8.7 percentage points [pp]; *P* < 0.01) in both EHFP groups compared with the control group (Olney et al., 2016). Children's haemoglobin (Hb; 5.1 g/L; *P* = 0.07), wasting (−8.8 pp; *P* = 0.08), and diarrhoea prevalence (−15.9 pp; *P* = 0.03) improved in the EHFP group who received BBC messages from health committee members compared with control; however, there were no observed improvements in child stunting or underweight (Olney et al., 2015). In the Nepal study, after over 2 years of intervention, anaemia and underweight were significantly lower for women who received EHFP (*OR*: 0.61; 95% CI [0.46, 0.82]; and *OR*: 0.62; 95% CI [0.48, 0.82], respectively; Osei et al., 2017). Anaemia was also significantly lower for children in the EHFP group (*OR*: 0.76; 95% CI [0.59, 0.98]) compared with control, but there was no observed impact on anthropometry in children (Osei et al., 2017). Although both are clearly well‐executed studies, neither study measured biochemical indicators of micronutrient status, beyond Hb, such as ferritin or zinc. There is also a need to evaluate EHFP in settings other than Western Africa or Nepal, where agricultural practices and diet likely differ and where anaemia rates and undernutrition are less severe.

Using a cluster‐randomized control trial design, the primary aim of this study was to evaluate the impact of an EHFP programme, with or without fishponds, on anaemia (Hb concentration measured by HemoCue*®*) in women and young children in Cambodia. The secondary outcomes were to investigate the impact of the EHFP programme on women's micronutrient status and women and children's anthropometry.

## 2 PARTICIPANTS AND METHODS

### 2.1 Study design

We used a cluster‐randomized controlled trial design. Ninety villages (clusters) in four districts (Kamchay Mear, Svay Anthor, Me Sang, and Bar Phnom) in Prey Veng province, Cambodia, were randomized to one of three treatments. Prey Veng is a densely populated agriculture and fishing region located in southeastern Cambodia bordering Vietnam and the east bank of the Mekong River (National Institute of Statistics Ministry of Planning, 2015). The project was approved by the Cambodian National Ethics Committee (010NECHR) and the Clinical Research Ethics board at the University of British Columbia (H12‐00451). Women provided informed consent for themselves and for their child prior to enrolment in the study. The trial was registered with http://clinicaltrials.gov as NCT01593423.

### 2.2 Randomization and allocation

Prey Veng was selected as the study province due to previously documented high rates of food insecurity and undernutrition (National Institute of Statistics, 2011; Santacroce, 2008). In 2008, Prey Veng was categorized as “chronically high food insecure” by World Food Program, with many households facing food deficits 3–4 months of every year (Santacroce, 2008). Anaemia in children 6–59 months and WRA was 47.8% and 41.2%, respectively (National Institute of Statistics, 2011). Of children 0–59 months, 35% were stunted (National Institute of Statistics, 2011).

Details of village and household selection have been reported elsewhere (Karakochuk et al., 2015; Verbowski et al., 2018). In summary, through key stakeholder consultation, HKI created a list of villages (*n* = 190) in Prey Veng that were not participating in any other food and nutrition interventions related to home gardening, poultry production, fish production, fruit production, infant and young child feeding education, or maternal nutrition activities. This formed the basis of the first shortlisting of villages in the four aforementioned districts that would be randomly selected for participation in the project. In total, 90 villages (clusters) were randomly allocated to one of three study arms: (a) plant‐based EHFP (homestead gardens only); (b) plant‐based EHFP plus fishponds (EHFP + F); or (c) control (no intervention). Subsequently, 10 households per cluster were randomly selected from the 90 clusters for a total of 900 households recruited. Random allocation was done by the study coordinator in Cambodia using a computer‐generated random number sequence in Excel.

In addition to technical training and input support for homestead gardens and fishponds, intervention households (EHFP and EHFP + F) received training on nutrition and hygiene, women's empowerment, and marketing. Further, one household per intervention village (*n* = 30 for the EHFP arm and *n* = 30 for the EHFP + F arm; total *n =* 60) was also selected to act as the village model farm (VMF) by HKI staff but was not considered part of the study sample.

### 2.3 Eligibility criteria

Households were eligible to participate in the trial if they were represented by a WRA (18–45 years) with a child 6–59 months, were considered “poor” according to national wealth rankings, and had access to “sufficient” land and labour to undertake homestead agriculture and polyculture activities. If more than one woman aged 18–45 years lived in the household, the primary caregiver (mainly mother) of the youngest child aged 6–59 months was selected. Households were identified as “poor” based on the Cambodian Ministry of Planning ID Poor classification, which ranks households based on observable and verifiable assets and sociodemographic traits, including school attendance, household composition, and the household dependency ratio (Cambodia Ministry of Planning, 2015). Households were said to have “sufficient” land if they had enough space for a 10 × 15‐m fishpond and an additional (undefined) small amount of land around the homestead for small‐scale plant‐based agriculture. The VMFs were selected based on the following criteria: (a) owned or had access to at least 1,200 m^2^ of homestead land for establishing a model farm; (b) had access to disposable income – at least 100 USD of available income – that could be used for VMF activities such as land preparation, fencing, housing for chicken, fish pond; (c) had suitable land for a pond near their homestead; (d) were able and willing to maintain year‐round gardening and polyculture activities; (e) were willing to provide technical assistance to study households in their respective village; and (f) committed to providing input support to village members during the duration of the project.

### 2.4 Interventions

All intervention households received basic agricultural inputs, such as seeds, seedlings, farming tools, and irrigation equipment (e.g. water pump and watering can), as required. Intervention households also received technical agricultural training and support and interpersonal BCC to target nutrition and hygiene practices and gender inequality. Households that were randomized to the EHFP + F group received assistance from the project to build new ponds or refurbish existing ponds, technical assistance for polyculture activities, and were provided with simple fish raising inputs such as fish nets, fish fry, and fingerlings. Additional details of the intervention packages can be found in Verbowski et al., 2018.

All inputs and training were implemented through VMFs. Specifically, VMFs acted as a community resource for agriculture and polyculture inputs and technical expertise for the study participants. Prior to implementation of project activities with all study households, VMFs received technical assistance, training, and simple input support from HKI staff and local NGO partners, including seeds, agricultural tools, and fingerlings. Once established, VMFs acted as a centre for training and technical assistance to enable study participants to establish their own EHFP activities. VMFs worked with HKI field officers to deliver technical group trainings on food production and ongoing ad hoc technical support to all households. In terms of inputs, VMFs provided a standard number of saplings and seedlings to all households at no cost as part of their cost‐sharing contribution to the project. All VMFs were fully established by October 2012.

Nutrition and Water, Sanitation, and Hygiene education focused on promoting the following practices based on the WHO's Essential Nutrition Actions framework: (a) optimal nutrition for pregnant and lactating women; (b) control and prevention of micronutrient deficiencies and anaemia; (c) optimal breastfeeding practices; (d) optimal complementary feeding practices; (e) optimal nutritional care of sick and malnourished children; and (f) essential hygiene actions (CORE Group & Nutrition Working Group, 2010; Guyon et al., 2009). Women's empowerment was addressed using a gender transformative approach adapted from the nurturing connections methodology developed by HKI in Bangladesh (Hillenband, Lindsey, Ridolfi, & Von Kotze, 2015). This approach identifies harmful gender norms and tries to change these norms to more positive and equitable practices. Gender messages were integrated into all project trainings, workshops, and meetings. Men were also included in nutrition training to remind them of the nutrition needs of their children and encourage them to share in childcare tasks with their wife. Regular monthly meetings were held where wives and husbands discussed issues related to gender disparities, especially those around decision‐making power and division of labour.

### 2.5 Procedures

Recruitment began in June 2012, and enrolment continued for 3 weeks. The trial was completed on July 10, 2014. Household surveys were conducted at baseline (June 2012) and at end line (May/June 2014), after 22 months of implementation. Hb concentration, weight, and height/length were measured for all women and children at baseline and at 22 months. Data were also collected on sociodemographic, homestead agriculture and fish production, food consumption, household food security, and on women and children's health, nutrition, and hygiene‐related knowledge, attitudes, and practices. Additionally, dietary intake data were collected from a subsample of *n* = 450 women and children. Details of dietary data collection procedures, assessment, and results are published elsewhere (Verbowski et al., 2018).

A finger prick capillary blood sample was taken from both women (18–45 years) and their youngest child (6–59 months) during household survey visits to measure Hb concentration using a portable photometer (HemoCue® Hb 201^+^). Anaemia for nonpregnant women was defined as Hb <120 g/L and for children aged 6–59 months as Hb <110 g/L. Weight and height/length were measured by trained research staff in accordance with established global guidelines (Cogill, 2003).

In addition to capillary blood, nonfasting venous blood was collected in a random subsample of *n =* 450 women at baseline and at 22 months. Three tubes were collected per woman: a trace element free tube (7.5 ml) and two Ethylenediaminetetraacetic acid (EDTA) coated tubes (2 and 10 ml). Tubes were stored in racks in ice boxes and transported daily to the National Institute of Public Health Laboratory in Phnom Penh. All precautions were taken to avoid zinc contamination; procedures for collection and analysis of serum zinc were in accordance with International Zinc Nutrition Consultative Group recommendations (Hotz & Brown, 2004). Serum ferritin, soluble transferrin receptor (sTfR), retinol binding protein (RBP), C‐reactive protein (CRP), and α‐1 acid glycoprotein (AGP) concentrations were measured at the VitMin Lab in Germany using an s‐ELISA. Vitamin B12 and folate were measured using an Abbott AxSym auto‐analyzer with appropriate controls and calibrators at the University of British Columbia. Serum zinc was analysed by flame atomic absorption spectrophotometry using a modified method of Smith, Butrimovitz, and Purdy (1979) at the University of Otago, New Zealand. Genetic Hb disorders were assessed using capillary Hb electrophoresis and PCR assays; venous blood collection and analysis procedures have been published elsewhere in full (Karakochuk et al., 2015).

Weight for women and children was measured using an electronic digital scale (Seca model 890, UNICEF, Copenhagen), to the nearest 100 g. Underweight women were defined as having a body mass index (BMI) <18.5 kg/m^2^. Sex and age‐specific *z*‐scores for children were calculated using the 2006 WHO child growth standards (WHO Multicentre Growth Reference Study Group, 2006), including height/length‐for‐age *z*‐scores, weight‐for‐height/length *z*‐scores, and weight‐for‐age *z*‐scores. Children were classified as stunted, wasted, or underweight if they had a height/length‐for‐age *z*‐scores, weight‐for‐height/length *z*‐scores, or weight‐for‐age *z*‐scores less than −2SD of the mean, respectively.

### 2.6 Outcomes

The primary outcome was the difference in the mean change in the prevalence of anaemia among nonpregnant women and their youngest child 6–59 months randomized to the EHFP or EHFP + F groups relative to control, using intention‐to‐treat analyses. The secondary outcomes were (a) the difference in the mean change of RBP, serum ferritin, sTfR, AGP, and CRP concentrations among nonpregnant women; (b) the adjusted mean difference in zinc concentrations among nonpregnant women at 22 months; and (c) the difference in the change in the proportion of underweight nonpregnant women, and stunted, wasted, and underweight children.

### 2.7 Statistical analyses

The number of clusters and households within each cluster was estimated based on the proportion of anaemic women and children, with 80% power and an a priori significant level of 0.025, to account for multiple comparisons. Assuming a 50% prevalence of anaemia and an interclass correlation of 0.05, a sample size of *n =* 300 for each arm provided 80% power to detect a 15% absolute reduction in the prevalence of anaemia.

Given the high attrition rates, instead of estimating missing data using multiple imputation, we employed the direct maximum likelihood method to account for the missing values at 22 months. The maximum likelihood method uses each respondent's available data to compute the likelihood function. The overall likelihood is the product of two factors: one computed for those respondents with missing data on some variables and the other for those with complete data on all variables. Parameter estimation and standard errors were derived from maximizing the overall likelihood function.

To describe the economic status of the study population, we applied a similar algorithm to the DHS (Rutstein & Johnson, 2004). In total, 21 indicator variables from the household survey questionnaires were selected to represent household economic status. Principal component analysis (PCA) was conducted, and the first principal component was extracted to generate a household asset score. This score was computed by multiplying factor weights (factor loading) with standardized original variables and used to create break points for the wealth index quintiles. PCA is considered a validated method to describe economic differentiation within a population (Filmer & Pritchett, 2001).

Baseline and 22‐month sample characteristics were summarized with means (*SD*) for continuous data and *n* (%) for categorical data. The distribution of the demographic characteristics across treatment groups was evaluated, and any demographic factor where differences across groups were deemed to be potential confounders based on potentially clinically importance was included in the final multivariate model. Generalized linear mixed effect models with repeated measures were used to evaluate the difference across groups and also the change from baseline to the end of the study (22 months). We included the interaction terms between group and time of visit, which represent the group differences in the change from baseline to 22 months, referred to as difference in difference (DID), with the exception of serum zinc for which baseline data were not collected. For DID estimates, data are presented as mean (*SE*) or pp. For serum zinc, data are mean difference (95% CI) at 22 months. The final models were adjusted for village clusters, wealth index quintiles, and women's educational attainment. The models of ferritin and sTfR were further adjusted for the Hb EE genotype, and children's Hb concentration were adjusted for the sex and age of the child. Ferritin and RBP values were also corrected for inflammation using AGP and CRP concentrations according to methods proposed by Thurnham, McCabe, Northrop‐Clewes, and Nestel (2003) and Thurnham et al. (2010). Women who were pregnant at baseline or at 22 months (determined by self‐reports during household surveys) were excluded from the biochemical or anthropometric analyses. Statistical analyses were performed using SAS 9.3 (SAS Institute Inc., Cary, NC). All reported *P* values are two sided.

## 3 RESULTS

### 3.1 Participant flow and loss to follow‐up

**Figure 1:**
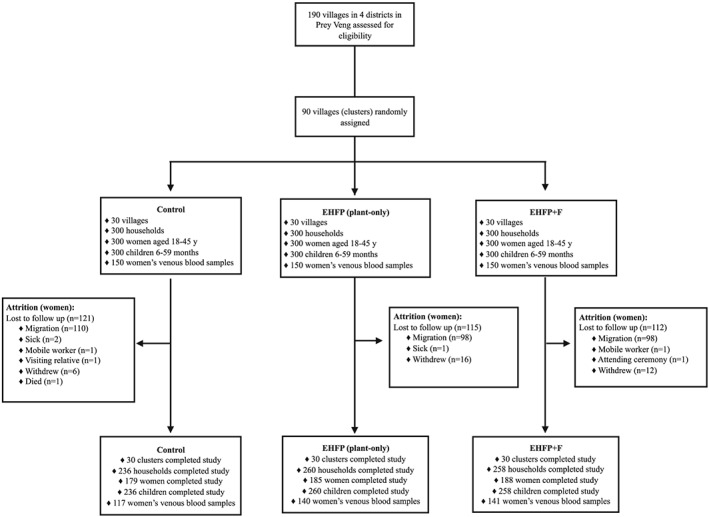
Participation flow and follow‐up. EHFP: enhanced homestead food production (plant‐based only); EHFP + F: enhanced homestead food production plus fishpond

Nine hundred households with a WRA and child (6–59 months) were enrolled in the study at baseline. At the end of the study, there were no missed clusters (*n* = 90). The overall household attrition rate at 22 months was 16.2% (*n* = 146) and did not differ across groups (*P* = 0.74; Figure 1). Attrition was higher for women only (38.6%; *n* = 348) than for households, mainly due to employment‐related temporary migration (Figure 1). Primary outcome data were available for *n* = 179, *n* = 185, and *n =* 188 women and *n* = 232, *n* = 255, and *n* = 245 children in the control, EHFP, and EHFP + F groups, respectively. Venous blood samples were successfully obtained from 88% of the subset of *n =* 450 women at 22 months (Figure 1). Loss to follow‐up for the venous blood draw was higher among women in the control group (22.0%, *n* = 33) than in the EHFP (6.7%, *n* = 10) and EHFP + F (6.0%, *n* = 9) groups.

### 3.2 Baseline characteristics

Household and participant characteristics are summarized in Table 1. Based on our evaluation of the demographic factors, it appeared that years of education and wealth quintiles were not equally distributed across groups; women on average had completed more years of schooling in the EHFP group than in the EHFP + F and control groups, and more households in the control group were in the bottom wealth index quintile as compared with households in the EHFP and EHFP + F groups. Therefore, these were included in the multivariable models as potential confounders.

**Table 1:** Baseline characteristics of all enrolled households, women (18–45 years), and children (6–59 months) in Prey Veng, Cambodia, by treatment group

	Control (*n =* 300)	EHFP (*n =* 300)	EHFP + F (*n =* 300)
Households			
Household size, mean (*SD*)	4.8 (1.6)	4.6 (1.5)	4.6 (1.5)
Wealth index quintiles, *n* (%)			
Lowest	78 (26.0)	53 (17.7)	49 (16.3)
Second	59 (19.7)	60 (20.0)	58 (19.3)
Middle	54 (18.0)	62 (20.7)	67 (22.3)
Fourth	67 (22.3)	51 (17.0)	58 (19.3)
Highest	42 (14.0)	74 (24.7)	68 (22.7)
Women			
Age, years, mean (*SD*)	29.6 (6.7)	29.8 (6.5)	29.4 (6.3)
Hb^a^, mean (*SD*)	121.5 (12.5)	121.7 (13.7)	122.4 (12.1)
Anaemia (Hb <120 g/L), *n* (%)	114 (41.0)	116 (41.9)	110 (39.0)
Underweight^a^ (BMI <18.5 kg/m^2^), *n* (%)	46 (16.6)	37 (13.4)	40 (14.2)
Parity (children born), *n* (%)			
1 child	108 (36.0)	98 (32.7)	110 (36.7)
2–3 children	142 (47.3)	148 (49.3)	145 (48.3)
≥4 children	50 (16.7)	54 (18.0)	45 (15.0)
Number of children <5 years, mean (*SD*)	1.2 (0.5)	1.3 (0.5)	1.2 (0.4)
Completed years of school, mean (*SD*)	3.8 (2.9)	4.6 (2.6)	3.8 (3.0)
Children^b^			
Boys, *n* (%)	156 (52.0)	163 (54.3)	167 (55.7)
Age, months, mean (*SD*)	24.3 (15.2)	24.4 (15.7)	24.2 (15.0)
Hb^c^, mean (*SD*)	105.7 (13.6)	104.1 (13.8)	104.5 (13.7)
Anaemia^c^ (Hb <110 g/L), *n* (%)	177 (59.2)	195 (65.4)	188 (63.1)
Stunted (HAZ less than −2SD), *n* (%)	88 (29.3)	68 (22.7)	83 (27.9)
Wasted (WHZ less than −2SD), *n* (%)	25 (8.3)	25 (8.4)	20 (6.7)
Underweight (WAZ less than −2SD), *n* (%)	69 (23.0)	78 (26.1)	70 (23.5)

*Note*. Values are mean (SD) or *n* (%). Wealth index is a weighted estimate of the economic status of a household based on ownership of assets (watch, motorcycle cart, ox‐cart, vehicle, CV/DVD player, and boat), type of housing materials (wall, roof, and floor), type of cooking fuel (charcoal, wood, electricity, natural gas, biogas, straw/shrubs/grass, and animal dung), access to electricity, ownership and size of agriculture land, ownership and size of homestead land, main source of drinking water (ring well, hand pump, tapped well, rain water, bought water, and hand dug), and ownership of livestock (cow/buffalo, pig, chicken, and duck). BMI: body mass index; EHFP: enhanced homestead food production (plant‐based only); EHFP + F: enhanced homestead food production (plant based) plus fishpond; Hb: haemoglobin; HAZ: length/height‐for‐age *z*‐scores; *SD*: standard deviation; WAZ: weight‐for‐age *z*‐scores; WHZ: weight‐for‐height *z*‐scores.

a Values exclude *n =* 63 pregnant women; control *n =* 278, EHFP *n =* 277, EHFP + F *n =* 282.

b Missing or invalid data for all children (*n* = 3); control *n* = 300, EHFP *n =* 299, EHFP + F *n =* 298.

c Missing data for children's Hb (*n* = 5); control *n* = 299, EHFP *n* = 298, EHFP + F *n* = 298.

Overall, mean (*SD*) maternal age was 29.5 (6.5) years. Women averaged between one and three children 6–59 months and *n* = 63 women were pregnant. The mean (*SD*) number of completed years of schooling was 4 (2.9) years and ranged from 0 to 14 years. In total, 41% of nonpregnant women were anaemic and 15% were underweight. The prevalence of anaemic, stunted, wasted, and underweight children was 63%, 27%, 8%, and 24%, respectively. Prevalence of micronutrient deficiencies and inflammation in the subsample of *n =* 450 women at baseline have been reported elsewhere (Karakochuk et al., 2015). Briefly, 7.4% (*n* = 31/450) of women had a Hb EE homozygous disorder, 2.1% (9/420) and 18.8% (*n* = 79/420) of nonpregnant women had iron deficiency, based on inflammation‐adjusted serum ferritin (<15 μg/L) and sTfR (>8.3 mg/L), respectively, with no evidence of folate (<3 μg/L), vitamin B12 (<150 pmol/L), or vitamin A deficiency (RBP <0.7 μmol/L).

### 3.3 Primary outcome

For nonpregnant women, we observed no statistically significant impact on Hb concentration between intervention and control groups (EHFP vs. control, *P* = 0.64; EHFP + F vs. control, *P* = 0.71; Table 2). Similarly, the change in anaemia rates from baseline to 22 months among nonpregnant women did not differ between intervention groups and control (EHFP vs. control, *P* = 0.55; EHFP + F vs. control, *P* = 0.87; Table 2). For children, however, we observed a statistically significant change in anaemia rates from baseline to 22 months in the EHFP group compared with control (−14.0 pp; *P* = 0.02) and a smaller, non‐significant reduction in the EHFP + F group compared with control (−9.74 pp; *P* = 0.12). Specifically, anaemia rates were 59.2%, 65.4%, and 63.1% at baseline and 59.5%, 52.6%, and 54.3% at 22 months for children in the control, EHFP, and EHFP + F groups, respectively. A similar trend was seen for Hb concentration among children in both intervention groups compared with control, although did not reach statistical significance (DID for EHFP vs. control: 2.43 g/L; *P* = 0.088; and DID for EHFP + F vs. control: 2.54 g/L; *P* = 0.076).

**Table 2:** Unadjusted mean Hb concentration and anaemia prevalence at baseline and 22‐month and intent‐to‐treat DID impact analysis for those indicators among enrolled nonpregnant Cambodian women (18–45 years) and children (6–59 months), by treatment group

	Control	EHFP	DID	*P*	EHFP + F	DID	*P*
Nonpregnant women							
Hb, g/L							
Baseline	121.5 (12.5)	121.7 (13.7)	‐	‐	122.4 (12.1)	‐	‐
22 months	121.1 (12.1)	121.0 (11.9)	−0.63 (1.34)	0.637	122.9 (12.9)	0.49(1.33)	0.714
Anaemia (Hb <120 g/L)							
Baseline	109 (40.4)	113 (41.9)	‐	‐	105 (38.9)	‐	‐
22 months	60 (38.7)	70 (43.5)	4.14 pp	0.551	58 (35.8)	−1.10 pp	0.865
Children							
Hb, g/L							
Baseline	105.7 (13.6)	104.1 (13.8)	‐	‐	104.5 (13.7)	‐	‐
22 months	107.1 (12.9)	108.0 (12.3)	2.43 (1.42)	0.088	108.4 (13.1)	2.54(1.43)	0.076
Anaemia (Hb <110 g/L)							
Baseline	177 (59.2)	195 (65.4)	‐	‐	188 (63.1)	‐	‐
22 months	138 (59.5)	134 (52.6)	−14.0 pp	0.023	133 (54.3)	−9.74 pp	0.119

*Note*. All unadjusted baseline and 22‐month values are mean (*SD*) or *n* (%). DID estimates between groups with logit function for binary data, from generalized linear mixed effect models adjusted for village clusters, years of education, and household wealth index values; data are presented as mean (*SE*) or percentage point (pp). Children's models were further adjusted for the sex and age of the child. Values exclude *n =* 89 pregnant women, with *n =* 270, 270, and 271 in control, EHFP and EHFP + F groups, respectively. Total number of children (6–59 months) included in analysis: *n* = 895 and *n* = 5 were excluded due to missing values. DID: difference in difference; EHFP: enhanced homestead food production (plant‐based only); EHFP + F: enhanced homestead food production (plant based) plus fishpond; Hb: haemoglobin; pp: percentage points; *SD*: standard deviation; *SE*: standard error.

### 3.4 Secondary outcomes

In the subset of nonpregnant women with a venous blood sample, we observed statistically higher mean ratio RBP concentrations at 22 months in the EHFP + F group compared with control group (*P* = 0.04), but no difference among women in the EHFP versus control (*P* = 0.49; data not shown). Further, we observed a statistically significant change in mean RBP concentration from baseline to 22 months for women in the EHFP + F versus control groups (DID: 0.34; *P* = 0.016; Table 3). Specifically, RBP concentration remained stable for the women in the EHFP + F group from baseline to 22 months, but we observed a considerable drop in RBP concentration for women in the control group (Table 3). No other statistically significant differences were observed for women's biochemical and inflammation biomarkers, ferritin, sTfR, serum zinc, AGP, or CRP between groups at 22 months (Tables 3 and 4); however, it is important to note that we did not collect baseline serum zinc, and therefore could not adjust for differences that might have been present at baseline.

**Table 3:** Unadjusted mean ferritin, soluble transferrin receptor, retinol binding protein, C‐reactive protein, and α‐1 acid glycoprotein concentrations at baseline and 22‐month and intent‐to‐treat DID impact analysis for those indicators among enrolled nonpregnant Cambodian women (18–45 years), by treatment group

	Control	EHFP	DID	*P*	EHFP + F	DID	*P*
Serum ferritin, μg/L							
Baseline	99.6 (56.6)	97.1 (52.5)	‐	‐	88.9 (62.1)	‐	‐
22 months	109.0 (68.5)	97.8 (52.1)	−11.68 (8.31)	0.161	100.1 (57.2)	−1.22 (8.08)	0.879
sTfR, mg/L							
Baseline	6.9 (2.5)	6.9 (7.9)	‐	‐	7.2 (2.8)	‐	‐
22 months	6.7 (2.2)	6.7 (2.3)	0.02 (0.36)	0.958	7.3 (3.4)	0.06 (0.35)	0.867
RBP, μmol/L							
Baseline	2.5 (0.7)	2.6 (0.7)	‐	‐	2.5 (0.7)	‐	‐
22 months	2.1 (0.7)	2.3 (0.9)	0.10 (0.14)	0.487	2.5 (1.0)	0.34 (0.14)	0.016
CRP, mg/L							
Baseline	1.7 (3.4)	2.5 (6.3)	‐	‐	1.7 (2.8)	‐	‐
22 months	1.6 (3.1)	1.1 (1.1)	−1.34 (0.75)	0.075	1.9 (2.1)	0.20 (0.73)	0.788
AGP, g/L							
Baseline	0.8 (0.3)	0.9 (0.3)	‐	‐	0.9 (0.3)	‐	‐
22 months	0.8 (0.4)	0.8 (0.2)	−0.05 (0.05)	0.255	0.8 (0.2)	−0.04 (0.05)	0.407

*Note*. Unadjusted baseline and 22‐month values are mean (*SD*). DID estimates between groups from generalized linear mixed effect models with gamma distribution and logarithmic link function for skewed data adjusted for village clusters, years of education, and household wealth index values; data are presented as mean (*SE*). The models of ferritin and sTfR were further adjusted by Hb EE genotype variant. Ferritin and RBP values were corrected for inflammation using AGP and CRP according to methods by Thurnham et al. (2003, 2010). Pregnant women excluded (*n =* 40), with *n* = 135, 137, and 138 in control, EHFP, and EHFP + F groups, respectively. AGP: α‐1 acid glycoprotein; CRP: C‐reactive protein; DID: difference in difference; EHFP: enhanced homestead food production (plant‐based only); EHFP + F: enhanced homestead food production (plant based) plus fishpond; RBP: retinol binding protein; *SD*: standard deviation; *SE*: standard error; sTfR: soluble transferrin receptor.

**Table 4:** Unadjusted mean serum zinc concentration at 22 months and difference in adjusted mean (95% CI) serum zinc concentrations at 22 months among enrolled nonpregnant Cambodian women (18–45 years), by treatment group

	Control	EHFP	EHFP + F	EHFP/control	EHFP + F/control
	Mean (*SD*)	Mean (*SD*)	Mean (*SD*)	Mean difference (95% CI)	Mean difference (95% CI)
Serum zinc, μmol/L					
22 months	11.0 (2.5)	10.8 (1.7)	10.8 (1.7)	−0.17 (−0.74, 0.40)	−0.16 (−0.73, 0.41)
*P* value	‐	‐	‐	0.66	0.67

*Note*. Baseline serum zinc concentrations were not assessed. Total *n* = 398 and *n =* 12 pregnant women were excluded. Mean difference between groups from generalized linear mixed effect models adjusted for village clusters, years of education, and household wealth index values. CI: confidence intervals; EHFP: enhanced homestead food production (plant‐based only); EHFP + F: enhanced homestead food production (plant based) plus fishpond; *SD*: standard deviation.

Lastly, anthropometric indicators of nutritional status for women and children were compared between treatment groups (Table 5). We observed no statistically significant impact on underweight in nonpregnant women (*P* > 0.05) or stunting, wasting, and underweight in children (*P* > 0.05).

**Table 5:** Unadjusted prevalence of underweight nonpregnant women (18–45 years), and stunted, wasted, and underweight children (6–59 months) at baseline and 22‐month and intent‐to‐treat DID impact analysis for those indicators

	Control *n* (%)	EHFP *n* (%)	DID	*P*	EHFP + F *n* (%)	DID	*P*
Women's BMI, kg/m^2^							
Baseline	46 (16.6)	37 (13.4)	‐	‐	40 (14.2)	‐	‐
22 months	16 (9.4)	24 (13.5)	4.27 pp	0.347	16 (9.0)	1.19 pp	0.920
Children's anthropometry							
Stunting (HAZ less than −2SD)							
Baseline	88 (29.3)	68 (22.7)	‐	‐	83 (27.9)	‐	‐
22 months	73 (32.0)	72 (28.9)	3.73 pp	0.453	72 (29.9)	−0.62 pp	0.927
Wasting (WHZ less than −2SD)							
Baseline	25 (8.3)	25 (8.4)	‐	‐	20 (6.7)	‐	‐
22 months	17 (8.9)	27 (13.0)	3.80 pp	0.348	20 (10.2)	2.75 pp	0.424
Underweight (WAZ less than −2SD)							
Baseline	69 (23.0)	78 (26.1)	‐	‐	70 (23.5)	‐	‐
22 months	66 (28.8)	72 (28.8)	−3.63 pp	0.479	77 (32.0)	2.75 pp	0.670

*Note*. Unadjusted baseline and 22‐month values are *n* (%). DID estimates between groups with logit function for binary data, from generalized linear mixed effects models adjusted for village clusters, years of education, and household wealth index values; data are presented as percentage point (pp). Values exclude *n =* 89 pregnant women, with *n =* 270, 270, and 271 in control, EHFP and EHFP + F groups, respectively. Total number of children (6–59 months) included in analysis: *n* = 897 and *n* = 3 were excluded due to missing values, with *n =* 300, 299, and 298 in control, EHFP, and EHFP + F, respectively. BMI: body mass index; DID: difference in difference; EHFP: enhanced homestead food production (plant‐based only); EHFP + F: enhanced homestead food production plus fishpond; HAZ: height/length‐for‐age *z*‐score; pp: percentage points; WAZ: weight‐for‐age *z*‐score; WHZ: weight‐for‐height *z*‐score.

## 4 DISCUSSION

This study aimed to rigorously examine the effects of an EHFP programme on anaemia and the nutritional status of poor Cambodian women farmers and their young children. To our knowledge, this is the largest published cluster‐randomized controlled trial of an integrated small‐scale agriculture and polyculture programme, with multiple measures of maternal and child nutritional status, including Hb concentration, anthropometry, and biochemical measures of micronutrient status.

Compared with controls, we observed a statistically significant reduction in anaemia rates in children in the EHFP group (*P* = 0.02) and a similar non‐significant reduction in anaemia among children in the EHFP + F group. We suspect that the decline in anaemia rates among children in the EHFP + F group did not reach statistical significance (*P* = 0.13) due simply to a few more children crossing the “cut‐off” used to define anaemia (<110 g/L) in the EHFP group and thus classified as nonanaemic. Both intervention groups had similar increases in mean Hb concentration (~2.5 g/L) compared with control, however, the EHFP+F group did not reach our criterion that is conventionally used to define statistical significance (*P* < 0.05) but would have met Olney et al. (2015) criterion for “marginal significance” (*P* < 0.10).

The change in anaemia status among children in the intervention groups compared with control is consistent with published findings from EHFP programmes conducted in Burkina Faso and Nepal (Olney et al., 2015; Osei et al., 2017). In Burkina Faso, a 5.1 g/L increase in Hb concentration (*P* = 0.06) was observed in children (3–12.9 months) who were randomized to the EHFP group who received BCC messages from health committee members compared with controls (Olney et al., 2015). Further, when the children were disaggregated by age, the authors found a larger and statistically significant effect in younger children aged 3–5.9 months, with a 7.6 g/L (*P* = 0.02) increase in Hb concentration and a 14.6% (*P* = 0.02) reduction in anaemia prevalence (Olney et al., 2015). In Nepal, by the end of the study, the authors reported a significantly lower anaemia rate for children in the EHFP group compared with control (30.8% vs. 42.5%, respectively; Osei et al., 2017). Similar to the authors of the Nepal and Burkina Faso studies, we do not have sufficient data to attribute this increase in Hb concentration or anaemia reduction to one particular aspect of the EHFP programme. Potential explanations include improvements in nutrient status, such as iron, or a reduction in inflammation. Whether these are mediated through direct or indirect pathways (i.e., increased nutrient intake or uptake of the BCC on improved nutrition and hygiene practices) is not known.

In comparison to children's anaemia rates, we found no impact of our interventions on maternal anaemia status. One reason for this discrepancy may be due to the fact that there appears to be a low prevalence of iron, folate, and vitamin A deficiency, and a high prevalence of genetic Hb disorders in Cambodian woman enrolled in our trial (Karakochuk et al., 2015). This is in contrast to the Burkina Faso study, where it is believed that iron and other micronutrient deficiencies are large contributors to the extremely high rates of anaemia in women (Magalhaes & Clements, 2011). The lack of iron and vitamin A deficiency in WRA, while a relatively new result, has been reported in nationally represented surveys across almost all provinces in Cambodia (National Institute of Statistics et al., 2015; Wieringa et al., 2016). In the 2014 Cambodia DHS, only 3% of WRA from a nationally representative sample were found to be iron deficient based on inflammation‐adjusted serum ferritin (<15 μg/L), <1% were vitamin A deficient, and approximately 60% of women were found to have a genetic Hb disorder (National Institute of Statistics et al., 2015; Wieringa et al., 2016). A subsequent study conducted in Kampong Chhnang province screened 2,846 WRA age for anaemia, of whom 809 were screened as anaemic and enrolled in an iron supplementation trial (Karakochuk et al., 2017). At baseline, only 19% and 22% of women were iron deficient anaemic, based on inflammation‐adjusted ferritin and sTfR, respectively (Karakochuk et al., 2017). Given the low prevalence of iron and other micronutrient deficiencies associated with anaemia in women and the high prevalence of genetic Hb disorders, we speculate that a nutrition‐sensitive agriculture‐based programme would not be an effective intervention for maternal anaemia reduction in this population.

We found improved vitamin A status, based on RBP concentration, in women randomized to the EHFP + F group compared with control. These findings are consistent with research conducted by Verbowski et al. (2018) that found, on the same sample, women in the EHFP + F group had significantly lower prevalence of inadequate vitamin A intake (66% vs. 47%; *P* < 0.05) and higher mean intake of vitamin A in both intervention groups (EHFP alone or EHFP + F) compared with control. However, given that no women had an RBP concentration indicative of deficiency at baseline (inflammation adjusted RBP concentration <0.7 μmol/L), the clinical significance of this finding is questionable.

The lack of impact on maternal underweight, and stunting, wasting, and underweight in children observed in the current study is not surprising. First, because anthropometry was only a secondary outcome, the study was likely underpowered to detect a small difference in stunting. Specifically, previous research suggests large sample sizes are required (~*n* = 1,200 per arm) to detect even a 20% difference in stunting if the baseline prevalence is at least 40%, and even larger if the prevalence is lower, as observed in the current study (Herforth & Ballard, 2016). Second, at baseline, children in the current study were on average 24 months old, and by the end of the study, on average 41 months; thus, the intervention would be less likely to improve anthropometry in this age group, as after 2 years, impact on growth faltering is limited. Third, to our knowledge, there has yet to be any published nutrition‐sensitive agriculture programme to demonstrate a statistically significant effect on underweight, wasting, and stunting in children (Ruel et al., 2013). Similarly, very few studies have shown a significant effect on maternal underweight, with the exception of the previously mentioned Burkina Faso and Nepal studies (Girard et al., 2012; Olney et al., 2016; Osei et al., 2017). This may be partially explained by the short duration and inadequate sample sizes of most studies (Herforth & Ballard, 2016; Masset et al., 2012), as well as the complex aetiology of undernutrition, particularly stunting.

Finally, we observed no additional beneficial effect of EHFP + F over EHFP alone with respect to anaemia in either women or children. For the secondary biochemical outcomes in women, we may have been underpowered; however, the mean differences in biochemical outcomes were small and inconsistent, sometimes favouring EHFP over EHFP + F. A larger sample size is very unlikely to have yielded different results. Based on our findings, we would not recommend fishponds over animal husbandry such as poultry, in this context.

Strengths of this study are the rigorous cluster‐randomized control trial design and the assessment of numerous outcomes along the programme impact pathways to improved nutritional status, namely, nutritional biochemical indicators of micronutrient status and anaemia. There are, however, a number of limitations that may have influenced the results of the study. First, we only controlled for the presence of the Hb EE homozygous genotype in women in our trial, as it has shown to be the strongest negative predictor of maternal Hb in the study population (Karakochuk et al., 2015); however, we acknowledge that there are other known genetic Hb disorders that may have affected biochemical results (e.g., Hb E heterozygous or α‐thalassemia). Second, attrition was higher than expected during the project, perhaps in part because of better livelihood opportunities for women in other geographical areas that resulted in an employment‐related temporary relocation and also because women were not offered a choice in the type of EHFP activity that they wished to participate in. As women were not able to select their preferred livelihood intervention, it is possible that the impact on nutrition, food security, and other indicators could be underestimated as participants may not have had the interest or motivation to undertake the EHFP activity. Further, the project provided many inputs “cost free,” and we speculate that the women may not have been as invested as they might have been otherwise. Research has shown that participants have a greater sense of ownership and stake in new technologies and practices, with better outcomes, when demand and type of intervention is driven by participants and when their own investment is higher than donations received (Prokopy, 2005; Sara & Katz, 1998; Stein, 2001). Lastly, the study was limited to only one province in Cambodia, and only participants who were poor and had access to land were eligible to participate, limiting the generalizability of the results to the wider population. However, given that the majority of households in Prey Veng own or have access to homestead land for small‐scale agriculture and fish production, land access likely did not limit household participation.

In summary, this cluster‐randomized controlled trial of EHFP in Cambodia showed improvements in childhood anaemia and a clinically insignificant increase in vitamin A status but no other differences in biochemical or anthropometric measures of women or children's nutritional status. It is possible that a longer duration of intervention may have produced greater improvements in anaemia and other biochemical indicators where dietary intake was shown to improve in the same study sample (Verbowski et al., 2018). There is a need for future nutrition‐sensitive agriculture programmes to assess changes in nutritional status in women and children over a longer period of time (e.g., 5–10 years) and include women who are pregnant, to cover the period of greatest vulnerability, particularly when assessing the impact on stunting, as changes are likely to be intergenerational and may not appear during the first 2 years of an intervention. In addition, households, particularly women, need to be invested and convinced of the monetary and health benefits at the outset of such programmes, perhaps even investing their own money, and be provided with the option to choose the types of agriculture or small‐scale animal‐husbandry activities to adopt. Without investment in the programme, the potential impacts will most likely be underestimated, and the attrition rates are likely to be high, as observed in the current study.

## CONFLICT OF INTEREST

KD Michaux, K Hou, CD Karakochuk, KC Whitfield, S Ly, V Verbowski, A Stormer, K Porter, KH Li, LA Houghton, LD Lynd, A Talukder, J McLean, and TJ Green declare no conflicts of interest. The funders had no role in the study design, data collection and analysis, decision to publish, or preparation of the manuscript.

## CONTRIBUTIONS

TJG, AT, LDL, and JM designed the research; CDK, KCW, VV, and SL conducted the research; SL and KH oversaw implementation and data management; AS and KP provided operational support; KHL conducted statistical analyses; KDM drafted the manuscript; all authors contributed to data interpretation and manuscript revision; KDM and TJG had primary responsibility for final content; all authors read and approved the final manuscript.
